# Eligibility of patients with chronic obstructive pulmonary disease for inclusion in randomised control trials investigating triple therapy: a study using routinely collected data

**DOI:** 10.1186/s12931-024-02672-x

**Published:** 2024-01-18

**Authors:** Hannah R. Whittaker, Aria Torkpour, Jennifer Quint

**Affiliations:** 1 School of Public Health, Imperial College London, London, UK; 2 Imperial College School of Medicine, Imperial College London, London, UK

**Keywords:** COPD, RCT, Real world evidence

## Abstract

**Background:**

Randomised control trials (RCTs) with strict eligibility criteria can lead to trial populations not commonly seen in clinical practice. We described the proportion of people with chronic obstructive pulmonary disease (COPD) in England eligible for RCTs investigating treatment with triple therapy.

**Methods:**

MEDLINE and Clinicaltrials.gov were searched for RCTs investigating triple therapy and eligibility criteria for each trial were extracted. Using routinely collected primary care data from Clinical Practice Research Datalink Aurum linked with Hospital Episode Statistics, we defined a population of COPD patients registered at a general practice in England, who were ≥ 40 years old, and had a history of smoking. Inclusion date was January 1, 2020. Patients who died earlier or left the general practice were excluded. Eligibility criteria for each RCT was applied to the population of COPD patients and the proportion of patients meeting each trial eligibility criteria were described.

**Results:**

26 RCTs investigating triple therapy were identified from the literature. The most common eligibility criteria were post-bronchodilator FEV_1_% predicted 30–80%, ≥ 2 moderate/≥ 1 severe exacerbations 12-months prior, no moderate exacerbations one-month prior and no severe exacerbations three-months prior, and the use of maintenance therapy or ICS use prior to inclusion. After applying each RCT eligibility criteria to our population of 79,810 COPD patients, a median of 11.2% [interquartile range (IQR) 1.8–17.4] of patients met eligibility criteria. The most discriminatory criteria included the presence exacerbations of COPD and previous COPD related medication use with a median of 67.6% (IQR 8.5–73.4) and 63% (IQR 69.3–38.4) of COPD patients not meeting these criteria, respectively.

**Conclusion:**

Data from these RCTs may not be generalisable to the wider population of people with COPD seen in everyday clinical practice and real-world evidence studies are needed to supplement trials to understand effectiveness in all people with COPD.

**Supplementary Information:**

The online version contains supplementary material available at 10.1186/s12931-024-02672-x.

## Introduction

Following national and international guidelines, COPD treatments are prescribed using a stepped approach, with individuals starting on the least intense treatment and progressing to a stronger treatment if the current treatment is ineffective [1, 2]. COPD patients who do not have asthmatic features or features suggesting steroid responsiveness should be offered dual therapy long-acting beta agonist (LABA) and long-acting muscarinic antagonist (LAMA) if they continue to experience poor health status on short-acting beta agonists (SABA) or short acting muscarinic antagonists (SAMA) [2]. NICE guidelines recommend stepping up from LABA/LAMA dual therapy to triple therapy consisting of LABA/LAMA and an inhaled corticosteroid (ICS) if the patient has one severe or more than 2 moderate exacerbations of COPD per year on dual therapy.

The use of triple therapy in people with COPD has been comprehensively studied through randomised control trials (RCTs) and has been associated with a reduced risk of future COPD exacerbations, improvement in lung function, symptoms, and health status compared with ICS/LABA, LABA/LAMA or LAMA monotherapy [1, 3, 4]. Guidelines for COPD management and treatment are predominantly based on results from randomised controlled trials (RCTs), as this is generally considered to be the optimal study design to test the efficacy and safety of medical interventions [5]. RCTs require a well-characterised patient population, and stringent selection means that findings may be limited in the extent to which treatment effects can be extrapolated to a broad general patient population for whom these treatments are ultimately prescribed [6, 7]. We know for example, that people with COPD in routine clinical practice tend to be older than trial participants, and people with multiple co-morbidities are often excluded from inclusion in clinical trials [8, 9]. Therefore, it is possible that the benefits of treatments identified in RCTs may not be favourable to patients not studied.

There is a widespread and frequently quoted assumption that over 90% of people treated for COPD would be ineligible to participate in RCTs [10–12]. However, epidemiological studies are needed to investigate this further to determine how populations studied in RCTs compare to the wider population of people seen in clinical care. Therefore, we first aimed to determine the most common inclusion and exclusion criteria for RCTs investigating the use of triple therapy in people with COPD. Second, we aimed to apply these common inclusion and exclusion criteria to a population of COPD patients using routinely collected electronic healthcare record data from England and describe the proportion of COPD patients that would be eligible for RCTs investigating triple therapy.

## Methods

### Selection of RCTs

First, we conducted a literature review to identify RCTs investigating the use of triple therapy (both fixed dose and combined inhalers) in people with COPD. Literature was searched through Medline and Clinicaltrials.gov. The following concepts were searched for: (i) chronic obstructive pulmonary disease; (ii) randomised control trial (including phase 3 and phase 4 trials). Additional file 1: Table S1 reports the full list of search terms used. Literature from the 1st of January 2012 was searched. Studies from 2012 onwards were included to capture studies that were published following a major GOLD 2011 guidelines update [13]. Additionally, studies were included if they compared triple therapy with other long term COPD maintenance therapies. Specifically, this included the comparison of any LABA/LAMA/ICS medications with any LABA, LAMA, LABA/LAMA. This was because in routine clinical practice patients are prescribed COPD medication in a stepwise fashion, with individuals starting on the least intense treatment and progressing to a stronger treatment if the current treatment is ineffective. Studies were excluded if they compared triple therapy with placebo, if the study population included non-COPD patients, and if the study included patients younger than 40 years old. RCT study names and key study variables were extracted, including the criteria that were used to enrol participants to these trials. These variables were categorised into inclusion and exclusion variables.

### Study population

Data from the Clinical Practice Research Datalink (CPRD) Aurum were linked to secondary care data from Hospital Episode Statistics (HES) and Index of Multiple Deprivation (IMD). CPRD Aurum contains routinely collected data from general practices across England and are representative of the English population in terms of geographical area, deprivation, age and sex [14].

**Fig. 1:**
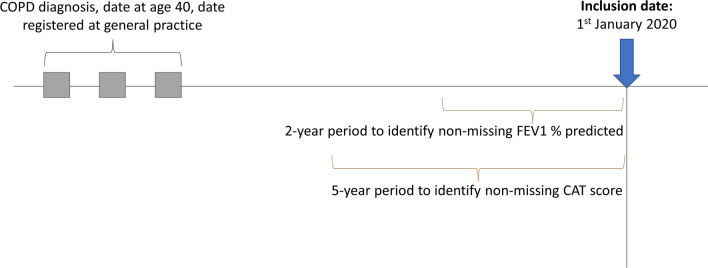
Study design. *COPD* chronic obstructive pulmonary disease, *FEV1* forced expiratory volume in 1 s, *CAT* COPD Assessment Test

Using these data, we defined a population of people who had been diagnosed with COPD in primary care using SNOMED CT codes, who were over the age of 40 years old, and who were registered at a general practice in England (Fig. 1). The date at which patients satisfied these criteria was the date at which they were eligible for the study. The date at which inclusion and exclusion criteria were applied was the 1st of January 2020 after patients met the eligibility criteria. Patients were excluded if they died or left their general practice earlier than the 1st of January 2020. Due to missingness of data in CPRD Aurum, all patients were required to have complete data on all variables of interest. Therefore, patients with missing forced expiratory volume in 1 s (FEV_1_) in the two years prior to inclusion date and missing COPD Assessment Test (CAT) score data in the 5 years prior to the inclusion date were excluded (Fig. 1).

### Definition of inclusion and exclusion variables

Inclusion and exclusion criteria identified from the literature search were applied to our cohort of COPD patients. All criteria were applied prior to index date. Variables that involved tests, such as lung function, were defined in the two years prior to index date. Variables that involved a history of a disease were defined at any time prior to index date. Medication based variables that were described as current were defined as a recorded prescription in the four months prior to index date. Current asthma was defined as having a diagnosis of asthma within the two years prior to a COPD diagnosis following a previous study [15]. A history of asthma was defined as having a diagnosis of asthma recorded prior to the definition of current asthma. Asthma variables were defined prior to first COPD diagnosis rather than index date due to possible misdiagnosis of asthma after a COPD diagnosis. A history of cardiovascular disease (CVD) was defined as having a recorded diagnosis of myocardial infarction, stroke, heart failure, or ischemic heart disease prior to index date. All other variable definitions were based on those extracted from RCTs in the literature search (Additional file 1: Table S2). Only the most common inclusion and exclusion criteria identified from RCTs in the literature search were applied to the cohort of COPD patients.

### Statistical analysis

First, baseline demographics of the COPD study population were described in numbers and percentages or means and standard deviations. Baseline demographics included age, sex, IMD, record of an exacerbation in the year prior to index date, and FEV_1_ percent predicted (by GOLD grade) prior to index date. Second, we applied the inclusion and exclusion criteria from each RCT from the literature search to our COPD study population in a stepwise fashion. Numbers and percentages were reported at each stage. Third, we described the median proportion (and interquartile range) of patients who would have met each eligibility criteria. Specifically, we described the median proportion of COPD patients who would have met eligibility criteria based on FEV_1_% predicted, previous exacerbations of COPD, CAT score, prescribed medications prior to inclusion, history of asthma, and history of CVD. Lastly, as an exploratory analysis, we described the proportion of people meeting each study’s inclusion and exclusion criteria over time. Time was defined as the date at which each RCT was published.

## Results

A total of 89 studies met our literature search criteria. Of these, 26 RCTs met our search inclusion and exclusion criteria and were included in our final analysis (Additional file 1: Fig. S1). A summary of the included studies, including the most common inclusion and exclusion criteria, are reported in Table 1. RCTs that met the inclusion criteria included TRILOGY, TRINITY, FULFIL, IMPACT, KRONOS, TRIFLOW, ETHOS, TRIVERSYTI, COSMOS-J, INTREPID, TRIDENT, DARWiIN, and AIRWISE [16–27]. The most common inclusion criteria categories of the 26 studies included FEV_1_ percent predicted, exacerbations of COPD, CAT score and current medications. The most common exclusion criteria categories included exacerbations of COPD, medication history, and history of asthma.

**Table 1:** Summary of included RCT studies

Author	Study name or trial number	Population size	Mean age (SD)	% Females	Common inclusion criteria	Common exclusion criteria
Hoshino [40]	–	30	73.4	6.7	FEV_1_% predicted < 70	Current maintenance therapies, current asthma, CVD
Manoharan [41]	–	13	69	23.1	FEV_1_% predicted 30–80, current ICS/LABA use	Moderate ECOPD within 1 month or severe ECOPD within 3 months of inclusion
Lee [42]	NCT01397890	578	66.8	4.3	FEV_1_% predicted < 50, at least 1 moderate ECOPD in year prior to inclusion	Any severe ECOPD in month prior, ICS or OCS use 1 month prior to inclusion, history of asthma, history of CVD
Singh [16]	TRILOGY	1368	63.6	24.5	FEV_1_% predicted < 50, at least 1 moderate ECOPD in year prior to inclusion, CAT score ≥ 10, ICS/LABA, ICS/LAMA, LABA/LAMA or LAMA prescribed 2 months prior to inclusion	Any severe ECOPD in month prior to inclusion, current asthma and history of CVD
Vestbo [17]	TRINITY	2691	63.0	24	FEV_1_% predicted < 50, at least 1 moderate ECOPD in year prior to inclusion, CAT score ≥ 10, ICS/LABA, ICS/LAMA, LABA/LAMA or LAMA prescribed 2 months prior to inclusion	ECPOD 4 weeks prior to inclusion, on triple therapy 2 months prior to inclusion, current asthma and history of CVD
Sousa [43]	NCT02257372	236	64.2	33	FEV_1_% predicted < 70, current ICS/LABA use	ECOPD treated with antibiotics or oral corticosteroids within 6 weeks of inclusion, LAMA use within 1 week or LABA, LAMA/LABA use with 2 weeks of inclusion, current asthma, history of CVD
Lipson [18]	FULFIL	1810	63.9	26	FEV_1_% predicted < 50% or FEV_1_% predicted 50–80% with at least 2 moderate or 1 severe ECOPD in the year prior to inclusion, CAT score ≥ 10, current maintenance therapy	Current asthma
Lipson [19]	IMPACT	10,355	65.3	34	FEV_1_% predicted < 50% or FEV_1_% predicted 50–80% with at least 2 moderate or 1 severe ECOPD in the year prior to inclusion, CAT score ≥ 10, current maintenance therapy and ICS in last month	Current asthma
Ferguson [20]	KRONOS	1896	65.3	28.4	FEV_1_% predicted 25–80, CAT score ≥ 10, maintenance therapy (no monotherapy) 6 weeks prior to inclusion	At least 1 moderate ECOPD 6 weeks prior to inclusion or 1 severe ECOPD within 3 months of inclusion, current asthma
Dean [21]	TRIFLOW	22	64	59.1	FEV_1_% predicted 30–80, current ICS (dual or triple)	Moderate COPD within 2 months or a severe ECOPD within one year of inclusion
Ferguson [20]	NCT03478683 NCT03478696; two replicate studies	728	65.2	48.3	FEV_1_% predicted < 50% or FEV_1_% predicted 50–80% with at least 2 moderate or 1 severe ECOPD in the year prior to inclusion, maintenance therapy for more than 3 months prior to inclusion, CAT ≥ 10	Current asthma, history of CVD
Rabe [22]	ETHOS	8509	64.7	40.3	FEV_1_% predicted 25–65%, 1 ECOPD in year prior to inclusion if FEV_1_% predicted < 50% or 2 moderate or 1 severe if FEV_1_% predicted > 50%, CAT ≥ 10, at least 1 maintenance therapies in the month prior to inclusion	Current asthma
Salvi [28]	CTRI/2019/01/017156	396	61.1	4.9	FEV_1_% predicted 30–80, at least 2 ECOPD in year prior to inclusion	A moderate ECOPD 6 weeks prior or 1 severe ECOPD 3 months prior to inclusion, current asthma
Van den Berge [44]	NCT03836677	23	64.9	21.7	FEV_1_% predicted 30–80, bronchodilator use 3 months prior to inclusion	Moderate ECOPD within 3 months of inclusion, ICS use in 3 months prior to inclusion, current asthma
Zheng [23]	TRIVERSYTI	708	66	95.3	FEV_1_% predicted < 50, at least one ECOPD year prior to inclusion, dual maintenance therapy 2 months prior to inclusion	ECOPD in month prior to inclusion, current asthma
Bansal [45]	NCT03474081	800	66.2	32	FEV_1_% predicted 30–80, 2 moderate or 1 severe ECOPD in last year prior to inclusion if FEV1% predicted is 50–80%, CAT score ≥ 10, use of tiotropium in month prior to inclusion	Any ECOPD within 14 days of inclusion, no oral corticosteroids use in month prior to inclusion, current asthma
Saito [46]	NCT01751113	53	67.3	2	FEV_1_% predicted 30–75	Severe ECOPD in year prior to inclusion, oral corticosteroids use in month prior to inclusion, current asthma
Betsuyaku [47]	COSMOS-J	Protocol			FEV_1_% predicted 30–80	Oral corticosteroids use month prior to inclusion, current asthma
Papi [48]	TRIBUTE	1532	64.5	28	FEV_1_% predicted < 50, at least 1 ECOPD in the year prior to inclusion, CAT score ≥ 10, use of dual maintenance therapies 2 months prior to inclusion	Current asthma, history of CVD
Bremner [49]	NCT02729051	1055	66.3	26	FEV_1_% predicted < 50% or FEV1% predicted 50–80% with at least 2 moderate or 1 severe ECOPD in the year prior to inclusion, CAT score ≥ 10	Any ECOPD within 2 weeks of inclusion, asthma, history of CVD
Singh [16]	TRIDENT	178	62.7	33.1	FEV_1_% predicted 30–60, current ICS/LABA use prior to inclusion	Moderate ECOPD month prior to a severe ECOPD 3 months prior to inclusion, current asthma, history of CVD
Siler [50]	NCT01957163 & NCT02119286 : two replicate trials	619	63.7	64.3	FEV_1_% predicted ≤ 70	Severe ECOPD 3 months prior to inclusion, current asthma, history of CVD
Van der Palen [51]	NCT0298218	70	65	48	Fixed ICS/LABA therapy 4 weeks prior to inclusion	Current asthma
Worsley [﻿^25^]	INTREPID	Protocol			At least 1 ECOPD 3 years prior to inclusion, CAT score ≥ 10, use of maintenance therapies within 4 months of inclusion	ECOPD 2 weeks prior to inclusion
Clinical trials.gov	DARwiIN	Protocol			FEV_1_% predicted ≤ 60, at least 1 ECOPD within 1 year prior to inclusion, CAT score ≥ 10, use of ICS/LABA within 8 weeks of inclusion	Any ECOPD within 1 month of inclusion, current asthma, history of CVD
Clinical trials.gov	AIRWISE	Protocol			Current use of ICS, LABA, ICS/LABA prior to inclusion	Use of LABA/LAMA prior to inclusion, current asthma

Patient characteristics could not be extracted from articles that were RCT protocols

*FEV*_1_ forced expiratory volume in 1 s, *ECOPD* exacerbation of COPD, *ICS* inhaled corticosteroid, *LABA* long-acting beta agonist, *LAMA* long-acting muscarinic antagonist, *CAT* COPD Assessment Test, *CVD* cardiovascular disease

The most common threshold of FEV_1_ percent predicted used in the identified studies was post bronchodilator FEV_1_ percent predicted 30–80%. Where patients were required to have a CAT score, the most common threshold was ≥ 10. However, inclusion and exclusion criteria around exacerbations of COPD varied across studies. The most common exacerbation criteria used as an inclusion criterion was having at least 2 moderate exacerbations or at least 1 severe exacerbation in the year prior to inclusion. The most common exacerbation criteria used an exclusion criterion was having at least one moderate exacerbation in the month prior or at least one severe exacerbation in the 3 months prior to inclusion. In terms of medication use, 21 (81%) studies specified an inclusion criterion for use of medication and the most common criteria specified that patients were currently on maintenance therapy or ICS prior to inclusion.

### Application of RCT eligibility criteria to COPD population

**Fig. 2:**
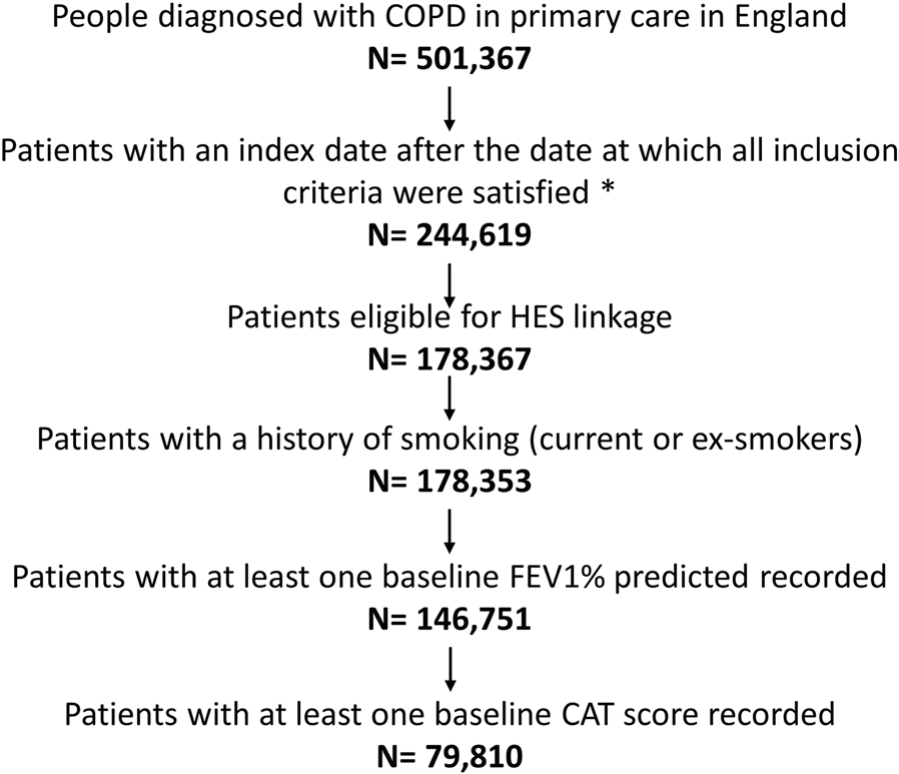
Inclusion of people to study.  *COPD* chronic obstructive pulmonary disease, *HES* hospital episode statistics, *FEV1* forced expiratory volume in 1 s, *CAT* COPD assessment test. *Inclusion criteria: aged older than 40, registered with a GP, diagnosis of COPD

A total of 178,367 people had a diagnosis of COPD, were over the age of 40, were registered at a GP in England and were eligible for HES linkage (Fig. 2). Of these people, the majority had a history of smoking and a total of 79,810 people had at least one baseline FEV_1_% predicted and CAT score recorded prior to index date.

In terms of baseline demographics, the mean age of the cohort of COPD patients was 71 years. Males represented 53.5% of the population and most patients were ex-smokers (62.4% of cohort). In addition, approximately half of patients had an FEV1% predicted between 50 and 80%. Mean CAT score was 14.7 and approximately one third of patients experienced an exacerbation of COPD in the year prior to inclusion, of which the majority were moderate exacerbations (Additional file 1: Table S3).

### Application of RCT inclusion and exclusion criteria to study population

**Fig. 3:**
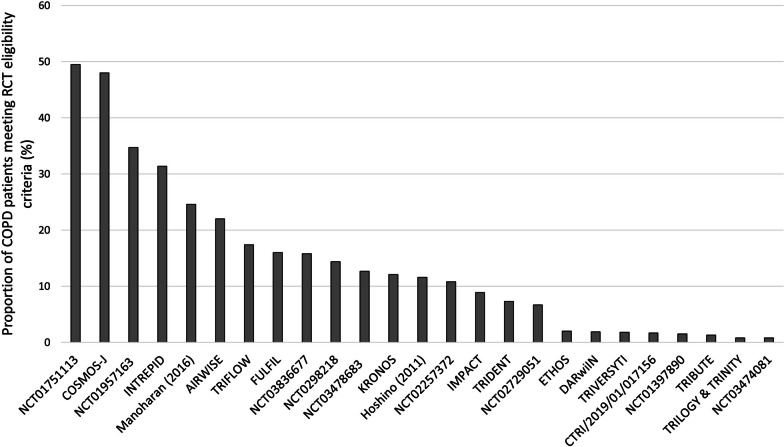
Proportion of people with COPD meeting RCT inclusion and exclusion criteria by RCT. *COPD* chronic obstructive pulmonary disease, *RCT* randomised control trial

The number of people who met each RCT’s eligibility criteria varied by RCT (Fig. 3). The proportion of patients meeting individual RCT criteria ranged from 0.8% to 49.5%. The median proportion of patients who met RCT eligibility criteria was 11.2% (IQR 1.8–17.4). Overall, 12 study’s inclusion and exclusion criteria led to the inclusion of fewer than 10% of all COPD patients in our population. In addition, 20 study’s inclusion and exclusion criteria led to the inclusion of fewer than 20% of all COPD patients in our population. Additional file 1: Table S4 illustrates the number of people meeting each of the inclusion and exclusion criteria per study.

**Fig. 4:**
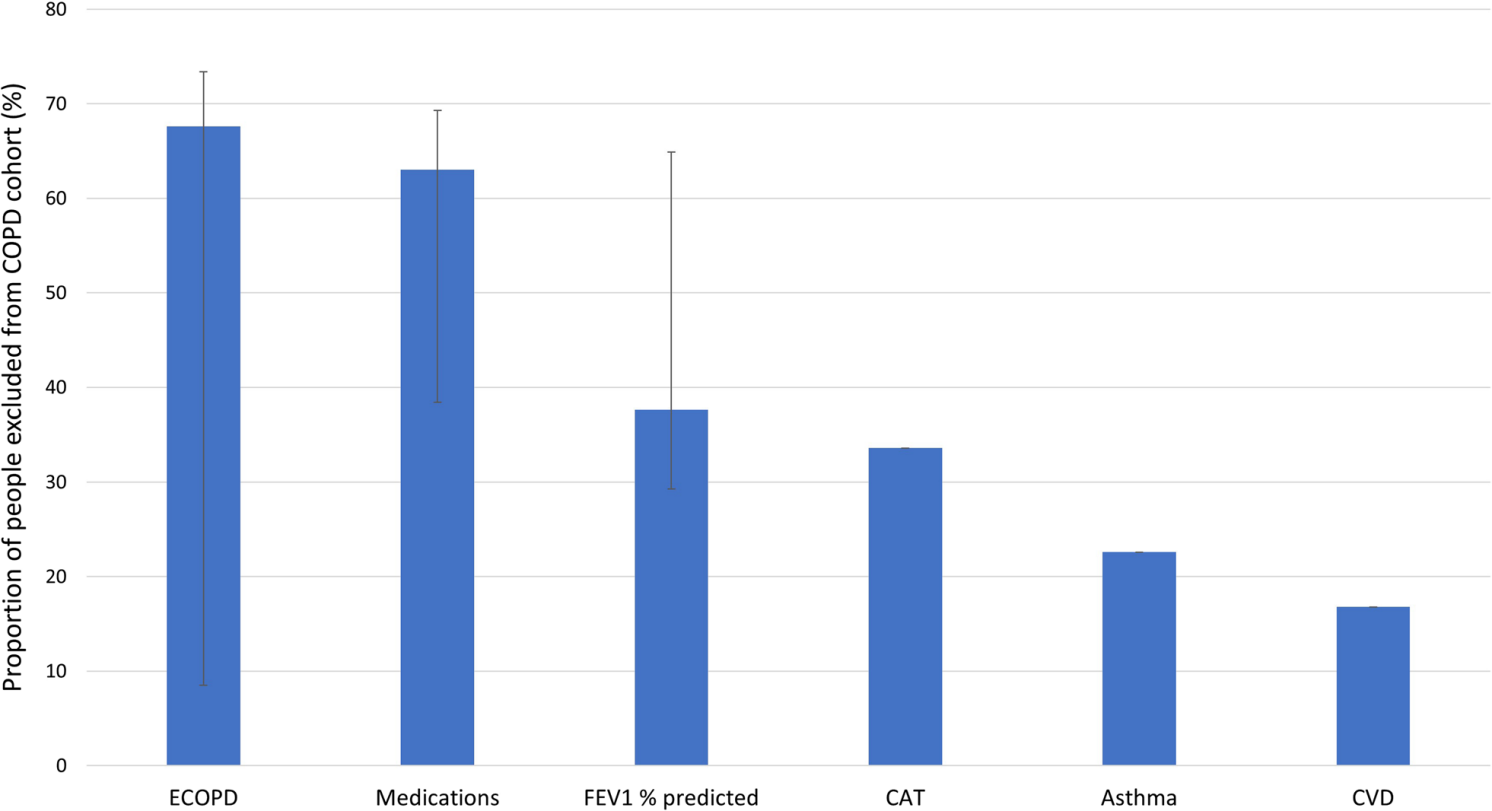
Median proportion of COPD patients excluded from COPD cohort based on domains of RCT eligibility criteria. Error bars are interquartile ranges. *ECOPD* exacerbations of COPD, *FEV1 *forced expiratory volume in 1 s, *CAT* COPD Assessment Test, *CVD* cardiovascular disease

The eligibility criteria that led to the fewest COPD patients included in our COPD population was around exacerbations of COPD. A median of 67.6% (IQR 8.5–73.4) of COPD patients would have been excluded based on exacerbation criteria alone (Fig. 4). The most discriminatory exacerbation-related inclusion criteria were having at least 2 exacerbations in the year prior to the inclusion date and no moderate exacerbation within 6 weeks and no severe exacerbations within 3 months of inclusion date [28]. Following this, the criteria that led to the second highest number of COPD patients excluded was based on previous or current prescribed medications where a total of 63% (IQR 38.4-69.3) of COPD patients would have been excluded based on medication criteria alone. The most discriminatory medication-related inclusion criterion was having at least 2 maintenance COPD therapies for at least 4 weeks prior to inclusion date [22]. Eligibility criteria around asthma and CVD resulted in the least COPD patients excluded.

When the proportion of COPD patients meeting RCT eligibility criteria was plotted over time, the proportion was lower for RCTs that were published more recently compared with studies published earlier however, no formal test was performed (Additional file 1: Fig. S2).

## Discussion

This study aimed to describe the proportion of COPD patients seen in routine clinical practice who would have met eligibility criteria for RCTs investigating triple therapy. Overall, we found that of the 26 RCTs that investigated triple therapy, the most common inclusion and exclusion criteria were based on lung function, exacerbations of COPD, current or history of prescribed medications, current or historical asthma, previous CVD, and CAT score. The average proportion of COPD patients who met each RCT eligibility criteria was 11.2% and the most common type of eligibility criteria was related to having exacerbations of COPD and current COPD medications. Specifically, the most discriminatory exacerbation-related inclusion criteria were having at least 2 exacerbations in the year prior to the inclusion date and no moderate exacerbation within 6 weeks and no severe exacerbations within 3 months of inclusion date. The most common discriminatory medication-related criteria were having at least 2 maintenance COPD therapies for at least 4 weeks prior to inclusion date. Further common eligibility criteria included a post bronchodilator FEV_1_ percent predicted 30–80%, having at least 2 moderate exacerbations or at least 1 severe exacerbation in the year prior to inclusion and no moderate exacerbations in the month prior and no severe exacerbations in the 3 months prior to inclusion, and the use of maintenance therapy or ICS use prior to inclusion. In addition, we found that fewer COPD patients met RCT eligibility criteria in more recent years.

Our findings are in keeping with a recent study that investigated the eligibility of COPD patients seen in routine clinical practice to RCTs investigating long-acting bronchodilator therapy [29]. Using data from the Optimum Patient Care Research Database (OPCRD), eligibility criteria from 31 RCTs were applied to a population of COPD patients. A median of 23% (IQR 12–38) of COPD patients met these inclusion and exclusion criteria. The median proportion of patients meeting eligibility criteria for RCTs investigating long-acting bronchodilator therapy was higher than the proportion of COPD patents meeting eligibility criteria for RCTs investigating triple therapy. Guidelines recommend triple therapy to COPD patients who experience at least one severe or two moderate exacerbations when on dual bronchodilator therapy [2]. This guideline is informed by RCTs which would have included COPD patients who exacerbated, and this is in line with findings from our study that found that previous exacerbations of COPD was the eligibility criteria that led to the fewest number of patients included.

A study by Pahus and colleagues investigated the proportion of COPD patients from the French Initiatives-BPCO database who would have met eligibility criteria from 16 RCTs where exacerbations was a primary outcome [30]. Overall, 2.3–46.7% of COPD patients met trial inclusion and exclusion criteria. The eligibility criteria that resulted in the exclusion of most people were based upon FEV_1_, previous exacerbations, and smoking history requirements. The most discriminatory eligibility criteria seen in our study varied slightly to those seen in the study by Pahus however, this is likely due to differences in the types of RCTs included in both studies. Whilst our study included eligibility criteria from RCTs investigating triple therapy, Pahus included RCTs that investigated a range of different types of therapies from dual bronchodilators to dual ICS/LABA to monotherapies. However, this study adds to the body of evidence highlighting the restrictive nature of trial populations and the lack of generalisable RCT populations.

Other studies have investigated the impact of eligibility criteria in RCTs in other disease areas including bronchiectasis and other chronic medical conditions. Using routinely collected data from centres in Scotland, England, Belgium, Italy and Ireland, one study found an average of 33% of people with bronchiectasis met the eligibility criteria of 10 bronchiectasis RCTs [31]. In addition, a further study using data from the Secure Anonymised Information Linkage (SAIL) Databank and data on participants included in over 116 RCTs found that the mean comorbidity count in the population of people included in trials was half of that seen in SAIL, a nationally representative population of people in Wales [9]. Other studies have found that fewer females are recruited to trials and trial populations are not representative in terms of age [32, 33]. Overall, our study, along with many others, highlights the lack of generalisability between populations studied in RCTs and populations seen in routine clinical practice.

Whilst RCTs will continue to remain the gold standard in assessing efficacy of intervention medical studies, the populations investigated are not always generalisable to the wider population of people seen in clinical practice. Results from trials should therefore only be extrapolated to the populations of people included in the study. Despite this, many people in clinical practice are prescribed medications regardless of the clinical indications. For example, studies have shown that ICS are overprescribed in clinical practice with approximately 50–80% of COPD patients prescribed ICS therapies and of these patients, very few meet the clinical indications for ICS prescription [34].One study found that as few as 10.6% of COPD patients on ICS-containing medications have a blood eosinophil count ≥ 300 cells/µl and a history of two or more moderate or one or more severe exacerbations in the previous year [35].

The risks of prescribing these medications in populations not studied in RCTs might not outweigh the benefits. ICS, for example, is associated with a higher risk of pneumonia and adverse events. Therefore, further studies are needed to determine whether results from trials can be extrapolated to other populations. One way to do this is through RCTs with less strict inclusion criteria and through real world evidence studies using large observational data to assess clinical effectiveness. Recently, the EMA and FDA have set up guidelines around the use of real-world evidence to inform health care related decisions which aims to monitor the effectiveness and safety of drugs post market [36, 37]. Studies using observational data to emulate RCTs using populations of people seen in clinical practice are starting to emerge and should continue to be used alongside RCTs to guide clinical guidelines for treatment of diseases and full and accurate reporting of trial selection criteria should be published manuscripts and in clinical trial databases [36, 38]. Furthermore, in RCTs people are required to discontinue current treatment at randomisation and switch to a treatment arm that can lead to an early effect of exacerbations or adverse events which may be due to abrupt change in treatment rather than the RCT treatment arm itself [39]. An adaptive RCT design which randomises on COPD treatment that is already being used by patients may also help to extrapolate results to wider populations. Additionally, RCT investigators could wait for disease-specific factors to resolve, such as exacerbations, prior to enrolment.

This study combines an extensive range of RCT selection criteria with a large, representative COPD patient population to provide detailed information on eligibility of patients with COPD for participation in RCTs. For example, if study criteria excluded people with asthma results should be interpreted based on the study population as it is possible that characteristics of people included played a role on the study findings. However, whilst data from routinely collected data sources can lead to more generalisable populations, there are limitations of the data that could have caused under or overestimated results in our study. First, we included people with COPD who had complete data on FEV1 and CAT scores prior to index date following a previous study [29]. It is possible that the total number of people meeting RCT inclusion and exclusion criteria could therefore be lower than expected as individuals with missing FEV1 or CAT could have been included in the final reported numbers. In theory these patients do have a FEV1 and a CAT score, but it was not recorded in the data and therefore they were excluded from our base population. This highlights the need for better recording of data in routinely collected data to effectively perform real world evidence studies. Second, we only applied the main inclusion and exclusion criteria to our base COPD population. There were other eligibility criteria that we didn’t apply due to lack of data availability and accuracy of the data in CPRD Aurum. This could have led to under or overestimation of the true proportion of COPD patients meeting RCT eligibility criteria. Lastly, the majority of RCTs excluded people based on “clinically important” comorbidities. However, these conditions are often not reported, and the definition of clinical importance can vary between clinicians. To minimise bias, we used the most common eligibility criteria and criteria that were clearly defined in the RCTs.

## Conclusion

Overall, very few COPD patients in routine clinical practice met eligibility criteria for RCTs investigating triple therapy. This was driven by inclusion and exclusion criteria around previous exacerbations and current COPD maintenance therapies. Whilst RCTs are essential in assessing the efficacy of medical interventions, results should only be extrapolated to the populations of people studied as the consequences of prescribing products beyond the population in which they were studied can result in unfavourable risk:benefit. Real world evidence studies are needed to supplement these studies to better understand clinical effectiveness in all types of COPD patients.

### Supplementary Information

**Additional file 1: Table S1.** Search terms. **Table S2.** Inclusion and exclusion criteria from RCTs. **Table S3.**: Baseline characteristics of cohort of COPD patients. **Table S4.** Number of COPD patients meeting each study’s main inclusion and exclusion criteria. **Figure S1.** Flow diagram of included studies. **Figure S2.** Proportion of COPD patients in study population meeting each RCT eligibility criteria over time.

## Data Availability

Data are available on request from the CPRD. Their provision requires the purchase of a license, and this license does not permit the authors to make them publicly available to all. This work used data from the version collected in May 2022 (10.48329/t89s-kf12) and have clearly specified the data selected within each Methods section. To allow identical data to be obtained by others, via the purchase of a license, the code lists will be provided upon request. Licenses are available from the CPRD (http://www.cprd.com): The Clinical Practice Research Datalink Group, The Medicines and Healthcare products Regulatory Agency, 10 South Colonnade, Canary Wharf, London E14 4PU.
