# Supplementary material for: Eligibility of patients with chronic obstructive pulmonary disease for inclusion in randomised control trials investigating triple therapy: a study using routinely collected data
Source: Respir Res. 2024 Jan 18;25:43. doi: 10.1186/s12931-024-02672-x (PMC10797743; doi:10.1186/s12931-024-02672-x)
Supplement: Supplementary file 1 — Additional file 1: Table S1. Search terms. Table S2. Inclusion and exclusion criteria from RCTs. Table S3.: Baseline characteristics of cohort of COPD patients. Table S4. Number of COPD patients meeting each study’s main inclusion and exclusion criteria. Figure S1. Flow diagram of included studies. Figure S2. Proportion of COPD patients in study population meeting each RCT eligibility criteria over time. [file 12931_2024_2672_MOESM1_ESM.docx]

**Eligibility of patients with chronic obstructive pulmonary disease for inclusion in randomised control trials investigating triple therapy: A study using routinely collected data**

Additional file

Contents

[Table S1: Search terms 2](#_Toc139029456)

[Table S2: Inclusion and exclusion criteria from RCTs 3](#_Toc139029457)

[Table S3: Baseline characteristics of cohort of COPD patients 14](#_Toc139029458)

[Table S4: Number of COPD patients meeting each study’s main inclusion and exclusion criteria. 15](#_Toc139029459)

[Figure S1: Flow diagram of included studies 17](#_Toc139029460)

[Figure S2: Proportion of COPD patients in study population meeting each RCT eligibility criteria over time. 18](#_Toc139029461)

# Table S1: Search terms

| Concept | Search Terms |  |
| --- | --- | --- |
| COPD | “COPD” | As text word in the abstract, title, subject heading, floating sub-heading, keyword heading, supplementary concept, protocol supplementary concept, rare disease supplementary concept, unique identifier, synonyms |
| Triple therapy | “Triple therapy” | As text word in the abstract, title, subject heading, floating sub-heading, keyword heading, supplementary concept, protocol supplementary concept, rare disease supplementary concept, unique identifier, synonyms, and name of substance |
| Study type | “Phase 3”, “Phase III”, “Phase 4”, “Phase IV “ | As text word in the abstract, title, subject heading, floating sub-heading, keyword heading, supplementary concept, protocol supplementary concept, unique identifier, synonyms |

# Table S2: Inclusion and exclusion criteria from RCTs

| **Author & year** | **Study name** | **Population size** | | | **Inclusion criteria** | | | **Exclusion criteria** |  |  |
| --- | --- | --- | --- | --- | --- | --- | --- | --- | --- | --- |
| Hoshino (2010) | - | 30 | | | - Age > 40 Years - Diagnosis of COPD - Cigarette smoking history > 10 pack-years - Post-Bronchodilator (BD) FEV1 < 70% predicted - FEV1:FVC < 0.70 | | | - Current diagnosis of asthma - Clinically significant medical disorder other than COPD - Supplemental oxygen use for exertion - Current use of respiratory medications (inc. ICS, LABA, Tio, theophylline or systemic corticosteroids) |  |  |
| Manoharan (2016) | - | 13 | | | - Age 40-80 years - Post-BD FEV1 30-80% predicted - Cigarette smoking history ≥ 10 pack-years - Current ICS/LABA use | | | - Other significant respiratory diseases - Moderate ECOPD within 1 month or severe ECOPD within 3 months of inclusion |  |  |
| Lee (2015) | NCT01397890 | 578 | | | - Age ≥ 40 years - Diagnosis of COPD with symptoms for > 2 years - At least 1 moderate ECOPD within 1-12 months of inclusion - Smoking history ≥ 10 pack-years - Pre-BD FEV1 ≤ 50% predicted - Pre-BD FEV1:FVC ratio < 0.70 | | | - A history of asthma - A history of seasonal allergic rhinitis before 40 years of age - Any severe ECOPD in month prior to inclusion - ICS or OCS use 1 month prior to inclusion - Significant CVD - Other significant respiratory tract disorder - Oral or ophthalmic use of NSBB - Use of Beta-blocking agents - Narrow angle glaucoma, benign prostatic hypertrophy or bladder neck obstruction |  |  |
| Singh (2016) | TRILOGY | 1368 | | | - Aged ≥ 40 years - Diagnosis of COPD - Post-BD FEV1 < 50% predcicted. - FEV1:FVC < 0.7 - At least 1 moderate/severe ECOPD in year prior to inclusion - CAT score ≥ 10 - BDI focal score ≤10 - Current use of ICS/LAMA, LABA/LAMA or LAMA prescribed 2 months prior to inclusion | | | - Current use of ICS/LABA/LAMA regime - Diagnosis of asthma - History of allergic rhinitis or atopy - Any ECOPD in month prior - Clinically significant history of CVD or laboratory abnormalities - Any unstable or concurrent disease that might affect efficacy or safety (judged by investigator) |  |  |
| Vestbo | TRINITY | 2691 | | | - Age ≥ 40 years - Current/ex-smokers - FEV1 < 50% predicted - FEV1:FVC < 0.70 - ≥ 1 ECOPD in past 12 months - Maintenance therapy on ICS/LABA or ICS/LAMA or LABA/LAMA, or LAMA 2 months prior to inclusion - CAT score>=10 | | | - ECPOD within a month of inclusion - Use of triple therapy 2 months prior to inclusion - Diagnosis of asthma - History of allergic rhinitis or atopy - Other respiratory conditions - History of CVD |  |  |
| Sousa (2016) | [NCT02257372](https://clinicaltrials.gov/ct2/show/NCT02257372) | 236 | | | - Age ≥ 40 years - Current/ex-smokers ≥ 10 pack-year history - Pre- and Post-BD FEV1 ≤ 70% - Pre- and Post-BD FEV1:FVC < 0.70 - mMRC Dyspnoea score ≥ 2 - Use of ICS/LABA for ≥ 1 month before inclusion | | | - Current diagnosis of asthma - Pneumonia or ECOPD treated with antibiotics or OCS within 6 weeks of inclusion - Lung volume reduction surgery within 12 months of inclusion - LRTI requiring antibiotic use within 6 weeks - Use of LTOT (>12 h/day) - Participation in acute phase of pulmonary rehabilitation programme 1 month from inclusion - Concurrent respiratory disease - Other clinically significant medical condition - Abnormal ECG finding at inclusion (CVD) - Use of prohibited medications |  |  |
| Lipson (2017) | FULFIL | 1810 | | | - Age ≥ 40 years - Diagnosis of COPD classified as GOLD group D:   - FEV1 < 50% predicted and CAT score ≥ 10   - FEV1 50-80% predicted and CAT score ≥ 10, and either 2 moderate or 1 severe ECOPD in year prior to inclusion - Current daily maintenance therapy for at least 3 months | | | - Current diagnosis of Asthma - Unresolved pneumonia or severe ECOPD |  |  |
| Lipson (2018) | IMPACT | 10,355 | | | - Age ≥ 40 years - CAT score ≥ 10 - FEV1 < 50% predicted or FEV1 50-80% predicted with at least 2 moderate or 1 severe ECOPD in the year prior to inclusion - Current maintenance therapy (inc. LABA, LAMA, or ICS alone or combined) before inclusion. | | | - Current diagnosis of asthma - Other significant medical conditions |  |  |
| Ferguson (2018) | KRONOS | 1896 | | | - Age 40-80 years - Current/ex-smokers ≥ 10 pack-year history - FEV1 25-80% predicted - CAT score ≥ 10 despite two or more maintenance therapies 6 weeks prior to inclusion | | | - Current diagnosis of asthma - Other respiratory conditions - 1 moderate ECOPD 6 weeks prior to inclusion or 1 severe ECOPD within 3 months of inclusion - Patients requiring spacer devices - Change in smoking status within 6 weeks of or during screening. - Use of LTOT (> 15h/day) |  |  |
| Dean (2020) | TRIFLOW | 22 | | | - Age 40-75 years - Post-BD FEV1 30-80% predicted - FEV1:FVC < 0.70 - RV > 120% predicted - Current/ex-smokers ≥ 10 pack-year history - Current ICS use (as part of dual or triple therapy) | | | - Other respiratory disorders - Moderate ECOPD within 2 months or a severe ECOPD within one year of inclusion - Significant abnormal clinical/laboratory or ECG finding (judged by investigator) |  |  |
| Ferguson (2020) | [NCT03478683](https://clinicaltrials.gov/ct2/show/NCT03478683) [NCT03478696](https://clinicaltrials.gov/ct2/show/NCT03478696); two replicate studies | 728 | | | - Age ≥ 40 years - CAT score ≥ 10 - FEV1 < 50% predicted or FEV1 50-80% predicted with at least 2 moderate or 1 severe ECOPD in the year prior to inclusion - Current maintenance therapy (inc. LABA, LAMA, or ICS alone or combined) before inclusion. | | | - Current diagnosis of asthma - Other significant comorbidities |  |  |
| Rabe (2020) | ETHOS | 8509 | | | - Age 40-80 years - CAT score ≥ 10 - At least 2 maintenance therapy in the month prior to inclusion - Post-BD FEV1 25-65% predicted:   - At least 2 moderate or 1 severe ECOPD in the year before screening if FEV1 ≥ 50% - Post-BD FEV1:FVC < 0.70 - Current/ex-smokers ≥ 10 pack-year history | | | - Current diagnosis of Asthma - History of asthma within past 5-10 years |  |  |
| Salvi (2021) | CTRI/2019/01/017156 | 396 | | | - Age 40-75 years - Current/ex-smokers ≥ 10 pack-year history - Post-BD FEV1 30-80% predicted - Post-BD FEV1:FVC < 0.70 - At least 2 ECOPD in year prior to inclusion - mMRC dyspnoea grade ≥ 2 - Use of ICS, ICS/LAMA, LABA/LAMA or LAMA maintenance therapies for at least 1 month before screening | | | - Asthma - A moderate ECOPD 6 weeks prior or 1 severe ECOPD 3 months prior to inclusion |  |  |
| Van den Berge | NCT03836677 | 23 | | | - Age 40-80 years of age - Current/ex-smokers ≥ 10 pack-year history - FEV:FVC < 0.70 - Post-BD FEV1 30-80% predicted - Blood esoniphil count > 150 cells/mm^3^ - Bronchodilator use 3 months prior to inclusion | | | - Asthma - Other clinically significant disease other than COPD - Moderate ECOPD within 3 months of inclusion - ICS use in 3 months prior to inclusion |  |  |
| Zheng (2021) | TRIVERSYTI | 708 | | | - Age ≥ 40 years - Diagnosed with COPD ≥ 12 months - Current/ex-smokers ≥ 10 pack-year history - Post-BD FEV1 < 50% predicted - ≥ 1 exacerbations in previous 12 months - Maintenance therapy (excluding ICS/LABA/LAMA triple therapy) for ≥ 2 months | | | - Diagnosis of asthma - ECOPD in month prior to inclusion - Use of traditional Chinese medicines |  |  |
| Bansal (2021) | NCT03474081. | 800 | | | - Age ≥ 40 years - Current/ex-smokers ≥ 10 pack-year history - TIO only maintenance therapy for ≥ 3 months - Post-BD FEV1 < 50% predicted or FEV1 > 50% predicted and ≥ 2 moderate or ≥ 1 severe ECOPD in 12 months prior to inclusion - CAT score ≥ 10 | | | - Current diagnosis of asthma - Other respiratory conditions - Lung resection surgery in 12 months prior to inclusion - Pneumonia or any ECOPD within 14 days of inclusion - ≥ 1 month following last dose of oral/systemic corticosteroids - Unresolved RTI ≥ 7 days prior to inclusion - Change in COPD medication during run-in |  |  |
| Saito (2015) | [NCT01751113](https://clinicaltrials.gov/ct2/show/NCT01751113) | 53 | | | - Age 40-80 years - Current/ex-smokers > 10 pack-year history - Post-BD FEV1 30-75% predicted - Post-BD FEV1:FVC < 0.70 - mMRC Dyspnoea score ≥ 1 | | | - Diagnosis of asthma - Other respiratory disorders - Previous lung surgery - Current pulmonary rehabilitation - Use of LTOT (≥ 12 h/day) - Severe ECOPD in year prior to inclusion - Oral corticosteroid use in month prior to inclusion |  |  |
| Betsuako | COSMOS-J | 400 | | - Age 40-80 years - Current/ex-smokers > 10 pack-year history - Post-BD FEV1 30-80% predicted - Post-BD FEV1:FVC < 0.70 - mMRC Dyspnoea score ≥ 1 | | | | - Diagnosis of asthma - Other respiratory disorders - Lung transplant or resection surgery - Use of LTOT (≥12 h/day) - Plan to start or change to pulmonary rehabilitations programme during study - Regular use of systemic corticosteroids - Other significant/uncontrolled disease - Receiving other investigational drug within 4 weeks of study | | |
| Papi (2018) | TRIBUTE | 1532 | | | - Age ≥ 40 years - Current/ex-smokers - Post-BD FEV1:FVC < 0.70 - FEV1 < 50% predicted - At least 1 moderate or severe ECOPD in past 12 months - CAT score ≥ 10 - Using dual maintenance therapy for ≥ 2 months prior to inclusion | | | - Current diagnosis asthma with use of Inhaled/oral corticosteroids - Clinically significant CVD or laboratory abnormalities - Unstable concurrent disease (as judged by investigator) |  |  |
| Bremmer (2018) | [NCT02729051](https://clinicaltrials.gov/ct2/show/NCT02729051) | 1055 | | | - Age ≥ 40 years - Current/ex-smoker ≥ 10 pack-years - CAT score ≥ 10 - Post-BD FEV1:FVC < 0.70 - Post-BD FEV1 < 50% predicted and ≥1 moderate/severe ECOPD in previous 12 months, or Post-BD FEV1 50-80% predicted and ≥ 2 moderate or ≥ 1 severe ECOPD in past 12 months | | | - Current diagnosis of asthma - Alpha-1 antitrypsin deficiency - Active TB - Other respiratory disorders that were primary cause of symptoms - Lung resection in previous 12 months - Risk factors for pneumonia - Unresolved pneumonia or ECOPD within 2 weeks of inclusion |  |  |
| Singh (2016) | TRIDENT | 178 | | | - Age 40-70 years - Current/ex-smoker ≥ 10 pack-years - Post-BD FEV1:FVC < 0.70 - Post-BD FEV1 30-60% of predicted - Increase in FEV1 of ≥ 60mL at 30 mins of 80µg TIO inhalation - Current ICS/LABA use, or ICS/LABA/TIO < 1 month prior to inclusion | | | - Diagnosis of asthma - History of allergic rhinitis or atopy - Moderate ECOPD month prior to a severe ECOPD 3 months prior to inclusion - Hypersensitivity to any of the study drugs - Clinically significant laboratory or ECG abnormalities - Unstable concurrent disease - Use of LTOT (> 12h/day) |  |  |
| Siler (2015) | [NCT01957163](http://clinicaltrials.gov/show/NCT01957163) &  [NCT02119286](http://clinicaltrials.gov/show/NCT02119286) : two replicate trials | 619 | | | - Age ≥ 40 years - Current/ex-smoker ≥ 10 pack-years - Pre- and post-BD FEV1:FVC < 0.70 - FEV1 ≤ 70% predicted - mMRC dysnpoea score ≥ 2 | | | - Current diagnosis of asthma - Other respiratory conditions - Severe ECOPD 3 months prior to inclusion - Use of LTOT |  |  |
| Van der Palen (2018) | [NCT0298218](http://clinicaltrials.gov/show/NCT0298218) | 70 | | | - Age ≥ 40 years - Current/ex-smoker ≥ 10 pack-years - Fixed ICS/LABA +/- LAMA therapy for ≥ 4 weeks prior to inclusion | | | - Current diagnosis of asthma - Use of Ellipta, any capsule, Diskus or Turbuhaler within past 2 years - History of drug and alcohol misuse - Allergy to component of inhaler - Receipt of investigational drug/device ≤1 month prior to inclusion - Patients unable to read/complete a questionnaire |  |  |
| Worsley (2019) | INTREPID | - Age ≥ 40 years - CAT score ≥ 10 - Moderate to severe ECOPD in 3 years prior to inclusion - Current, non-ELLIPTA maintenance therapy for ≥ 16 weeks (inc. ICS/LABA/LAMA, ICS/LABA, LABA/LAMA) | | | | | - Unresolved ECOPD within 2 weeks of inclusion - Chronic use of oral corticosteroid - Pregnant, lactating women of child-bearing age - Medical conditions with low probability of 6 month survival - Historical or current uncontrolled or clinically significant disease. | | |  |
| Clinical trials.gov | DARwiiN | - Age ≥ 40 years - Current/ex-smoker ≥ 10 pack-years - Post-BD FEV1:FVC < 0.70 - Post-BD FEV1 ≤ 60% - Current non-extrafine ICS/LABA DPI maintenance therapy for ≥ 2 months from inclusion - CAT score ≥ 10 - ≥ 1 moderate/severe ECOPD within 12 months of inclusion | | | | - ECOPD within month of inclusion - Current diagnosis of asthma - Other Respiratory disorders - CVD - History of Lung transplant or resection surgery | | | |  |
| Clinical trials.gov | AIRWISE | | - Age ≥ 40 years - Current monotherapy with LABA or LAMA or ICS/LABA dual therapy | | | - Currently on LAMA/LABA (Free or FDC) or triple therapy - Contraindication to any of ICS, LABA or LAMA - Current diagnosis of asthma - Pregnancy or nursing women | | | |  |

| **Baseline characteristic** | **n (%) or mean (SD)** |
| --- | --- |
| Mean age | 71 (10.2) |
| Gender   Male  Female | 42,726 (53.5) 37,084 (46.5) |
| Smoking status  Ex-smoking  Current smoking | 49,782 (62.4) 30,028 (37.6) |
| Index of Multiple Deprivation  1 (most deprived)  2  3  4  5 (least deprived)  Missing | 11,269 (14.1) 13,689 (17.2) 14,251 (17.9) 17,499 (21.9) 23,067 (28.9) 35 (0.04) |
| FEV1 % predicted  ≥80%  50-80%  30-50%  <30% | 20,490 (25.7) 41,083 (51.5) 15,322 (19.2) 2,915 (3.7) |
| CAT score | 14.7 (22.9) |
| Exacerbation of COPD in year prior to inclusion  None  Any  At least one moderate*  At least one severe* | 54,041 (67.7) 25,769 (32.3) 22,240 (27.9) 6,719 (8.4) |

# Table S3: Baseline characteristics of cohort of COPD patients

*Legend: Numbers reported are proportions and percentages and means and standard deviations. SD (standard deviation), FEV1 (forced expiratory volume in 1 second), CAT (COPD Assessment Test), COPD (chronic obstructive pulmonary disease). *Numbers not mutually exclusive.*

Table S4: Number of COPD patients meeting each study’s main inclusion and exclusion criteria.
Legend: FEV1 (forced expiratory volume in 1 second), ECOPD (exacerbation of COPD), CAT (COPD Assessment Test), CVD (cardiovascular disease)

| Eligibility criteria domain | Study ID | | | | | | | | | | | | | |
| --- | --- | --- | --- | --- | --- | --- | --- | --- | --- | --- | --- | --- | --- | --- |
|  | **Hoshino (2010)** | **Manoharan (2016)** | **Lee (2015)** | **Singh (2016) & Vestbo (2017)** | **Sousa (2016)** | **Lipson (2017)** | **Lipson (2018)** | **Ferguson (2018)** | **Dean (2020)** | **Ferguson (2020)** | **Rabe (2020)** | **Salvi (2021)** | **Van den Berge (2021)** | **Zheng (2021)** |
| Base cohort | 79,810 (100) | | | | | | | | | | | | |  |
| FEV1 % predicted | 45,544 (57.1) | 56,405 (70.7) | 18,237  (22.9) | 18,237 (22.9) | 45,544 (57.1) | 59,320 (74.3) | 59,320  (74.3) | 57,939 (72.6) | 56,405 (70.7) | 59,320 (74.3) | 38,154 (47.8) | 56,405 (70.7) | 56,405 (70.7) | 18,237 (22.9) |
| ECOPD  *Inclusion* | - | - | 5,992 (7.5) | 7,506 (9.4) | - | 23,929 (30.0) | 13,198 (16.5) | - | - | 23,929 (30.0) | 10,588 (13.3) | 6,802 (8.3) | 50,219 (62.9) | 7,506 (9.4) |
| *Exclusion* | - | 52,045  (65.2) | 5,706 (7.5) | 6,063 (7.6) | 42,729 (53.5) | - | - | 52,670 (65.9) | 47,562  (59.6) | - | - | 4,051 (5.1) | - | 6,063 (7.6) |
| CAT | - | - | - | 4,994 (6.3) | - | 18,601 (23.3) | 10,551 (13.2) | 35,953 (45.0) | - | 18,601 (23.3) | 8,583 (10.8) | - | - | - |
| Medications   *Inclusion* | - | 19,663 (24.6) | - | 2,717 (3.4) | 17,016 (21.3) | 16,968 (21.3) | 9,689 (12.1) | 13,490 (16.9) | 24,700 (30.9) | 16,968 (21.3) | 2,261 (2.8) | 1,826 (2.3) | 26,244 (32.9) | 3,724 (4.7) |
| *Exclusion* | 12,777 (16.0) | - | 2,363 (3.0) | 1,091 (1.4) | 15,787 (19.8) | - | - | - | - | - | - | - | 13,740 (17.2) | 1,774 (2.2) |
| Asthma | 11,337 (14.2) | - | 1,872 (2.3) | 846 (1.1) | 10,385 (13.0) | 12,771 (16.0) | 7,137 (8.9) | 9,682 (12.1) | 16,465 (20.6) | 12,771 (16.0) | 1,570 (2.0) | 1,359 (1.7) | 12,573 (15.8) | 1,411 (1.8) |
| CVD | 9,247 (11.6) | - | 1,200 (1.5) | 646 (0.8) | 8,603 (10.8) | - | - | - | 13,858 (17.4) | 10,106 (12.7) | - | - | - | - |
| Total | **9,247 (11.6)** | **19,663 (24.6)** | **1,200 (1.5)** | **646 (0.8)** | **8,603 (10.8)** | **12,771 (16.0)** | **7,137 (8.9)** | **9,682 (12.1)** | **13,858 (17.4)** | **10,106 (12.7)** | **1,570 (2.0)** | **1,359 (1.7)** | **12,573 (15.8)** | **1,411 (1.8)** |

|  | Study ID | | | | | | | | | | |
| --- | --- | --- | --- | --- | --- | --- | --- | --- | --- | --- | --- |
| Eligibility criteria domain | **Bansal (2021)** | **Saito (2015)** | **Betsuyaku (2013)** | **Papi (2018)** | **Bremmer (2018)** | **Singh (2016)** | **Siler (2015)** | **Van der Palen (2018)** | **Wolsey (2019)** | **NCT04876677** | **NCT03265145** |
| Base cohort | 79,810 (100) | | | | | | | | | | |
| FEV1 % predicted | 56,405(74.3) | 49,824 (62.4) | 56,405 (70.7) | 18,237(22.9) | 59,320 (74.3) | 28,043 (35.1) | 45,544 (57.8) | - | - | 30,958 (38.8) | - |
| ECOPD  *Inclusion* | 21,014 (26.3) | - | - | 7,506 (9.4) | 13,198 (16.5) | - | - | - | 46,523 (58.3) | 11,882  (14.9) | - |
| *Exclusion* | 19,838 (24.8) | 45,068 (56.5) | - | - | 11,874 (14.9) | 25,446 (31.9) | 43,518 (54.5) | - | 44,432 (55.5) | 9,671 (12.1) | - |
| CAT | 15,192 (19.0) | - | - | 6,196 (7.8) | 9,512 (11.9) | - | - | - | 31,585 (39.6) | 7,688 (9.6) | - |
| Medications   *Inclusion* | 1,181 (1.5) | - | - | 3,707 (4.6) | - | 10,466 (13.1) | - | 18,080 (22.7) | 25,047 (31.4) | 2,875 (3.6) | 41,786  (52.4) |
| *Exclusion* | 921 (1.2) | 39,525 (49.5) | 49,029 (61.4) | 1,640 (2.1) |  |  |  |  |  |  | 23,423 (29.3) |
| Asthma | 653 (0.8) | - | 38,308 (48.0) | 1,299 (1.6) | 7,096 (8.9) | 6,952 (8.7) | 33,558 (42.0) | 11,485 (14.4) | - | 1,919 (2.4) | 17,595 (22.0) |
| CVD | - | - | - | 1,005 (1.3) | 5,338 (6.7) | 5,806 (7.3) | 27,692 (34.7) | - | - | 1,509  (1.9) | - |
| Total | **653 (0.8)** | **39,525 (49.5)** | **38,308 (48.0)** | **1,005 (1.3)** | **5,338 (6.7)** | **5,806 (7.3)** | **27,692 (34.7)** | **11,485 (14.4)** | **25,047 (31.4)** | **1,509  (1.9)** | **17,595 (22.0)** |

# Figure S1: Flow diagram of included studies


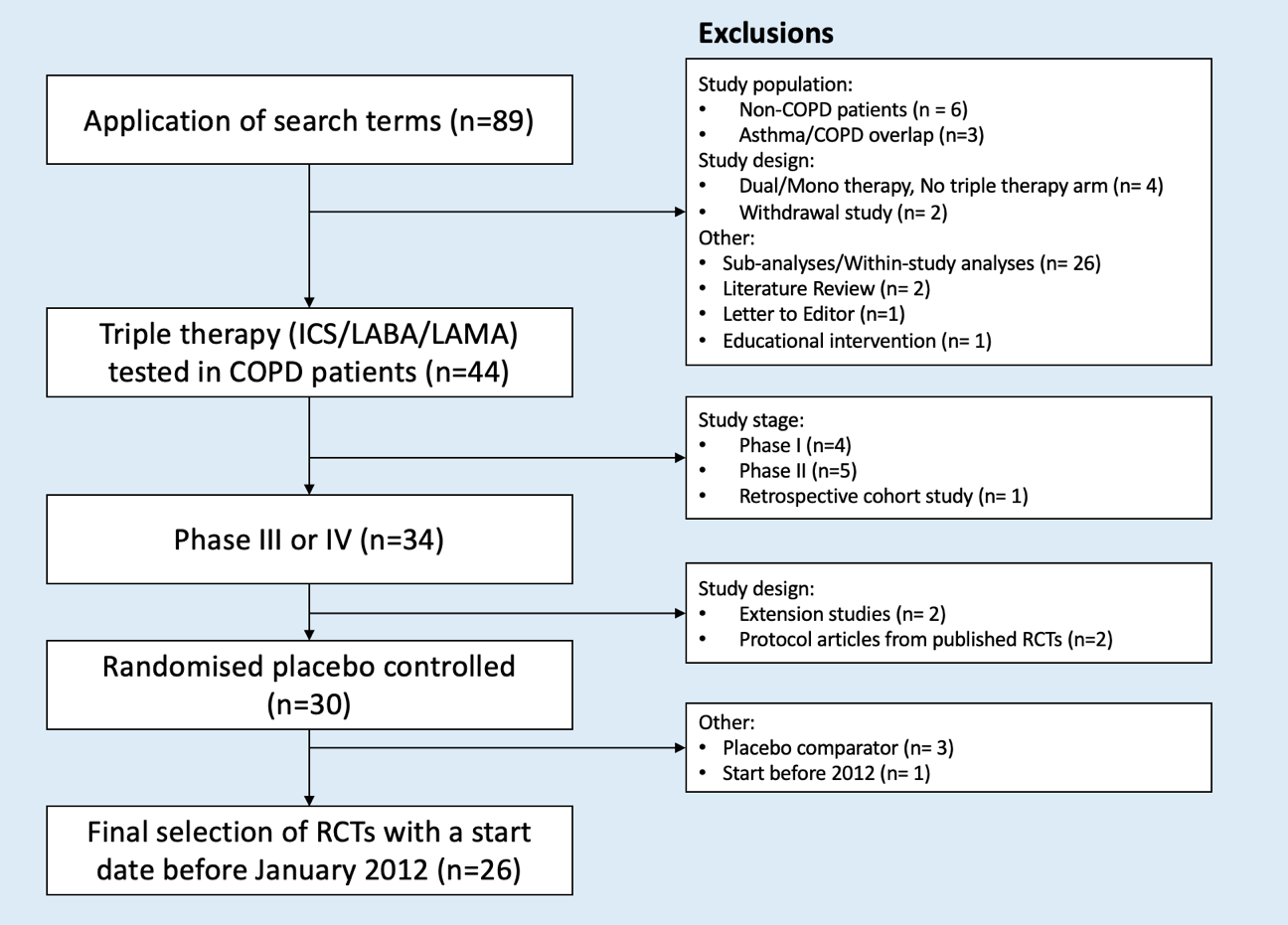


# Figure S2: Proportion of COPD patients in study population meeting each RCT eligibility criteria over time.
